# Data on the existence ratio and social utility of Nash equilibria and of the Perfectly Transparent Equilibrium

**DOI:** 10.1016/j.dib.2020.106623

**Published:** 2020-12-08

**Authors:** Ghislain Fourny, Felipe Sulser

**Affiliations:** a Department of Computer Science, ETH Zurich, Universitätsstrasse 6, Switzerland; b UBS AG, Zurich, Switzerland

**Keywords:** Strategic games, Game theory, Nash equilibrium, Perfectly Transparent Equilibrium, Social utility, Existence

## Abstract

This dataset includes 204,350,000 games in normal form played by two agents that have the choice between three strategies each, as well as 100,000 games in normal form played by four agents that have the choice between three strategies each. The games are in general position, i.e., there are no ties between the outcomes for each of the agents. These are simple random samples with replacement from the associated populations of strategic games with ordinal semantics. Each game was obtained with random permutations of the payoffs. Their Nash Equilibria as well Perfectly Transparent Equilibria are pre-computed. The existence ratios of the Nash equilibrium, unique Nash equilibrium and Perfectly Transparent Equilibrium were computed from the first sample with two players, and the social utilities from the second sample with four players.

**Specifications Table**

**Table:** 

Subject	Economics and Econometrics
Specific subject area	Game theory, games in normal form, Nash equilibrium
Type of data	JSON Lines (Zipped) JSON Lines (Text) CSV
How data were acquired	The games were randomly generated on a MacBook Pro (15 inch, 2016) with a Quad-Core Intel Core i7 processor, 2.6 GHz, 16 GB of RAM, using Python 2.7 and Spark 2.4.4. They were uploaded to Amazon S3. The existence ratios were computed with Amazon EMR 5.29, Hadoop 2.8.5 YARN, Spark 2.4.4, and Rumble 1.4.2 "Willow Oak" beta, on a cluster of m5.12xlarge machines, reading from Amazon S3.
Data format	Raw and partially analyzed data
Parameters for data collection	Games in normal form [1,2] in general position with ordinal semantics. Two or four agents. Three strategies per agent. Payoffs are permutations of the integers between 0 and 8 for two players, between 0 and 80 for four players. The Nash Equilibria [3] were pre-computed. The Perfectly Transparent Equilibria [4] were pre-computed. The games were obtained as simple random samples with replacement.
Description of data collection	This data was automatically generated with our Python library and PySpark. It was partially analyzed with JSONiq, Rumble and Apache Spark.
Data source location	ETH Zurich, Department of Computer Science, Switzerland
Data accessibility	Repository name: ETH Research Collection Data identification number: 20.500.11850/444921 Direct URL to data: https://www.research-collection.ethz.ch/handle/20.500.11850/444921 Repository name: ETH Research Collection Data identification number: 20.500.11850/444956 Direct URL to data: https://www.research-collection.ethz.ch/handle/20.500.11850/444956
Related research article	G. Fourny, Perfect Prediction in normal form: Superrational thinking extended to non-symmetric games, J. Math. Psychol. 96 (2020) 102332. https://doi.org/10.1016/j.jmp.2020.102332.

## Value of the Data

• The data is useful to acquire large-scale data-driven insights on games in normal form with ordinal semantics and in general position.
• Researchers in game theory and economics can use this data.
• Researchers can post-process the games to extend it with their own solution concepts and calculations, so as to formulate and test their hypotheses.
• The data reveals insights on the existence ratio of Nash equilibria, of unique Nash equilibria, and of Perfectly Transparent Equilibria depending on the degree of correlation of the agent payoffs.
• The data reveals insights on the social utility of unique Nash equilibria and of Perfectly Transparent Equilibria.

## 1 Data Description

Historically, game theory concepts have been designed with a theoretical and mathematical approach, completed with field experiments. With the considerable storage and computing power now available, a data-driven approach to game theory becomes feasible so as to develop and further understand solution concepts, old and new.

The first dataset provided here contains 204,350,000 games that constitute a simple random sample from the population of games in normal form [1,2] with two agents, three strategies each, in general position and with payoffs being integers between 0 and 8. The Nash Equilibria [3] are pre-computed as well as Perfectly Transparent Equilibria [4], but the dataset can be post-processed with an arbitrary number of other solution concepts.

The second dataset provided here contains 100,000 games that constitute a simple random sample from the population of games in normal form [1,2] with four agents, three strategies each, in general position and with payoffs being integers between 0 and 80. The Nash Equilibria [3] are pre-computed as well as Perfectly Transparent Equilibria [4], but the dataset can be post-processed with an arbitrary number of other solution concepts.

These datasets can be used to gain large-scale, data-driven insights on games and their solution concepts. They can be used to design new solution concepts, examine their existence, uniqueness, Pareto-optimality in a data-driven fashion, infer conjectures to support technical work, and to gain a deeper understanding on existing solution concepts. In particular, the datasets are made available together with partially analyzed data on the existence ratio and social utility of Nash equilibria vs. Perfectly Transparent Equilibria that were used to generate the plots in the related research paper.

The existence ratio CSV file available at [7] contains the data used to produce Figure 6 of this paper. The social utilities CSV file available at [8] contains the data used to produce Figure 15 of this paper. All other figures correspond to specific examples and are not tied to any dataset.

The first dataset is made available as five ZIP files containing each twenty JSON Lines files with each about 2 million games.

The second dataset is made available as eight JSON Lines files with each 12,000 or 13,000 games.

Each JSON Lines file name has the form "/part-00123.txt".

Each game is a JSON object on its own line.

The JSON syntax of a single game is shown on Table 1.

**Table 1:** JSON syntax of a two-player game, pretty-printed. In the dataset, each game appears on the same line.

{ "y": [ [ [7, 1], [4, 5], [3, 0] ], [ [1, 4], [0, 8], [6, 6] ], [ [2, 2], [5, 7], [8, 3] ] ], "P": [ [1, 2] ], "N": [ [2, 1] ] }

The game shown on Table 1 corresponds to the visual representation shown on Table 2, where the (in this case unique) Nash equilibrium appears in purple and the Perfectly Transparent Equilibrium appears in blue.

**Table 2:** Visual representation of a game that is part of the dataset, along with its Nash Equilibrium and Perfectly Transparent Equilibrium.

There are 204,350,000 two-player games in total. The organization in 100 JSON Lines files, typical of large-scale datasets, allows users to use subsets of arbitrary sizes depending on their needs, but also to run queries in parallel.

The structure of the 100,000 four-player games is similar, but has arrays of higher dimensionality to account for the higher number of players, and payoffs organized in quadruples, as shown on Table 3.

**Table 3:** JSON syntax of a four-player game, pretty-printed. In the dataset, each game appears on the same line.

{ "x": 17, "z": 4, "y": [ [[[[44, 75, 73, 69], [58, 77, 64, 64], [23, 56, 23, 24]], [[47, 70, 28, 15], [29, 24, 22, 53], [11, 2, 57, 35]], [[73, 43, 35, 63], [34, 21, 6, 9], [45, 13, 61, 27]]], [[[17, 69, 9, 70], [9, 30, 38, 20], [76, 65, 40, 43]], [[7, 4, 77, 16], [67, 7, 75, 32], [13, 78, 34, 37]], [[18, 10, 26, 23], [6, 80, 50, 40], [62, 9, 15, 29]]], [[[60, 6, 11, 54], [65, 12, 0, 21], [50, 66, 41, 26]], [[49, 63, 59, 74], [5, 49, 20, 12], [40, 51, 37, 61]], [[32, 41, 1, 56], [53, 40, 12, 59], [79, 57, 70, 72]]]], [[[[3, 64, 45, 5], [64, 53, 3, 31], [36, 35, 24, 57]], [[1, 28, 7, 55], [43, 34, 42, 67], [41, 22, 29, 58]], [[35, 59, 39, 60], [77, 1, 74, 11], [31, 46, 30, 38]]], [[[12, 17, 14, 44], [68, 5, 21, 4], [0, 31, 2, 77]], [[38, 52, 36, 75], [57, 16, 31, 73], [20, 23, 44, 78]], [[46, 72, 76, 52], [16, 73, 60, 62], [69, 76, 66, 1]]], [[[74, 15, 4, 45], [8, 39, 43, 6], [39, 18, 55, 18]], [[66, 19, 17, 80], [55, 25, 67, 19], [54, 26, 63, 22]], [[70, 20, 71, 79], [75, 33, 13, 48], [25, 0, 69, 30]]]], [[[[10, 37, 19, 33], [24, 29, 8, 34], [42, 44, 32, 41]], [[71, 62, 58, 25], [56, 14, 56, 65], [33, 32, 53, 39]], [[51, 8, 27, 3], [2, 60, 62, 7], [14, 55, 25, 50]]], [[[80, 38, 72, 0], [19, 74, 47, 42], [27, 68, 33, 36]], [[22, 42, 80, 46], [28, 11, 49, 2, 4, 27, 79, 8]], [[26, 71, 54, 10], [15, 48, 5, 14], [52, 47, 48, 76]]], [[[61, 79, 51, 49], [21, 50, 18, 13], [72, 36, 16, 68]], [[30, 3, 78, 51], [48, 58, 10, 47], [59, 67, 68, 17]], [[37, 61, 46, 66], [63, 45, 65, 28], [78, 54, 52, 71]]]] ], "P": [[0, 0, 0, 1]], "N": [[0, 2, 2, 2], [1, 1, 2, 1], [0, 0, 0, 0]] }

## 2 Experimental Design, Materials and Methods

The Python code used to generate the data is available at [5].

Each two-player game was generated by picking two random permutations of integers between 0 and 8, and using each of these permutations as the 9 payoffs of each of the two agents. These payoffs are put inside a three-dimensional array of integers stored in field "y".

Each four-player game was generated by picking four random permutations of integers between 0 and 80, and using each of these permutations as the 81 payoffs of each of the four agents. These payoffs are put inside a five-dimensional array of integers stored in field "y".

The Nash equilibrium was computed. Its zero-indexed coordinates in the two-dimensional array "y" are put in field "N". They consist of two integers between 0 and 2 for the two-player games, and of four integers between 0 and 2 for the four-player games (strategy indices).

The Perfectly Transparent Equilibrium was computed. Its zero-indexed coordinates in the two-dimensional array "y" are put in field "P". They consist of two integers between 0 and 2 for the two-player games, and of four integers between 0 and 2 for the four-player games (strategy indices).

The field "x" in the four-player sample is a numbering of the game and "z" indicates the number of players (four).

The CSV file containing data on the existence ratio was obtained as specified in [4], that is, by computing, for each game, the payoff correlation, by grouping the games in 81 payoff correlation buckets of size 0.025 between -1 and 1, and computing the ratio of existence of (i) at least a Nash Equilibrium, (ii) exactly one Nash Equilibrium and (iii) at least a Perfectly Transparent Equilibrium (which is then always unique).

The CSV file containing data on the social utilities was obtained as specified in [4], that is, by computing, for each game, the social utility of its unique Nash Equilibrium (if it exists) and of its Perfectly Transparent Equilibrium (if it exists). The social utility is obtained through the Theil-L function, rounded to the next integer. The games are then counted for each rounded social utility and for each equilibrium concept.

We recommend using this dataset with tools such as the JSONiq query language and the Rumble engine running on Apache Spark [6]. For faster performance, the dataset can also be stored on a distributed file system such as S3 or HDFS. This approach was taken in our research [4] and can be of interest to other researchers.

## Declaration of Competing Interest

The authors declare that they have no known competing financial interests or personal relationships which have, or could be perceived to have, influenced the work reported in this article.

## Appendix Supplementary materials

Image, application 1
